# Clinical trial of extended-dose chloroquine for treatment of resistant falciparum malaria among Afghan refugees in Pakistan

**DOI:** 10.1186/1475-2875-10-171

**Published:** 2011-06-23

**Authors:** Natasha Howard, Naeem Durrani, Sanda Sanda, Khalid Beshir, Rachel Hallett, Mark Rowland

**Affiliations:** 1 London School of Hygiene and Tropical Medicine (LSHTM), London, UK; 2 HealthNet-TPO, Peshawar Pakistan and Kabul Afghanistan

## Abstract

**Background:**

Falciparum malaria is a significant problem for Afghan refugees in Pakistan. Refugee treatment guidelines recommended standard three-day chloroquine treatment (25 mg/kg) for first episodes and extended five-day treatment (40 mg/kg) for recrudescent infections, based on the assumption that a five-day course would more likely achieve a cure. An *in-vivo *randomized controlled trial was conducted among refugees with uncomplicated falciparum malaria to determine whether five-day treatment (CQ40) was more effective than standard treatment (CQ25).

**Methods:**

142 falciparum patients were recruited into CQ25 or CQ40 treatment arms and followed up to 60 days with regular blood smears. The primary outcome was parasitological cure without recrudescence. Treatment failures were retreated with CQ40. PCR genotyping of 270 samples, from the same and nearby sites, was used to support interpretation of outcomes.

**Results:**

84% of CQ25 versus 51% of CQ40 patients experienced parasite recrudescence during follow-up (adjusted odds ratio 0.17, 95%CI 0.08-0.38). Cure rates were significantly improved with CQ40, particularly among adults. Fever clearance time, parasite clearance time, and proportions gametocytaemic post-treatment were similar between treatment groups. Second-line CQ40 treatment resulted in higher failure rates than first-line CQ40 treatment. CQ-resistance marker *pfcrt *76T was found in all isolates analysed, while *pfmdr1 *86Y and 184Y were found in 18% and 37% of isolates respectively.

**Conclusions:**

CQ is not suitable for first-line falciparum treatment in Afghan refugee communities. The extended-dose CQ regimen can overcome 39% of resistant infections that would recrudesce under the standard regimen, but the high failure rate after directly observed treatment demonstrates its use is inappropriate.

## Background

During the extended Afghan conflict, waves of refugees totalling almost three million entered northwest Pakistan and more than one million remain [1,2]. Malaria became a major problem in Afghan refugee camps, due to overstretched health infrastructure and some camps being located on marginal land prone to anopheline mosquito breeding [2]. By the 1990s, malaria among refugees increased ten-fold to over 100,000 cases per annum [2]. Approximately 30% of confirmed cases were due to *Plasmodium falciparum *and the remainder to *Plasmodium vivax *[3]. Chloroquine (CQ) was Pakistan's first-line treatment for uncomplicated falciparum malaria from 1950 to 2007 [3]. It remains first-line treatment for vivax malaria, so is still used for treating unconfirmed malaria and falciparum infections undetected by microscopy or misdiagnosed as vivax [2].

The United Nations High Commissioner for Refugees (UNHCR), following national guidelines, adopted a three-day CQ treatment course (total 25 mg/kg as 10 mg/kg on Day 0 and Day 1 and 5 mg/kg on Day 2) in refugee settlements. However, it became apparent during the 1990s that CQ was failing [4,5]. Basic health unit doctors claimed that many refugees stopped taking CQ tablets once clinical symptoms reduced or only took them intermittently. Health policy makers assumed that refugee patients were more likely to take sufficient CQ to cure infections if given a five-day course. Consequently, MoH Pakistan adopted as policy a five-day extended CQ course (CQ 40 mg/kg as 10 mg/kg/day on Days 0-2 and 5 mg/kg/day on Days 3-4) for any refugee patient returning to a basic health unit (BHU) with parasitaemia within a few weeks of their first episode. When this policy was introduced, no *in vivo *resistance survey had been undertaken in refugee camps, despite CQ-resistant falciparum parasites spreading widely in Pakistan in the 1990s [4-6].

As there was no evidence to support claims of poor adherence or the efficacy of extended-dose CQ, an open-label randomized clinical trial was conducted to determine whether supervised CQ treatment administered at 40 mg/kg over five days (CQ40) was more effective than 25 mg/kg over three days (CQ25) for curing infections completely without recrudescence [7]. The trial aim was to provide stronger evidence for the extended-dose CQ (ECQ) treatment or justification for discontinuing the policy.

## Methods

### Study design

The primary trial outcome was the proportion of individuals in each treatment arm that showed clinical and parasitological cure with no recrudescence. Sample size was calculated to detect a difference of 15% in cure rate between CQ25 and CQ40 treatment arms with 95% confidence and 90% precision, assuming a 20% loss to follow-up. The surveys were conducted during winter months to select only recrudescent episodes. Mosquito densities and malaria transmission drop during December and January, providing little opportunity for trial participants to receive further infective bites within the 60-day follow-up period [8,9]. Thus, subsequent falciparum episodes were regarded as recrudescence.

**Figure 1:**
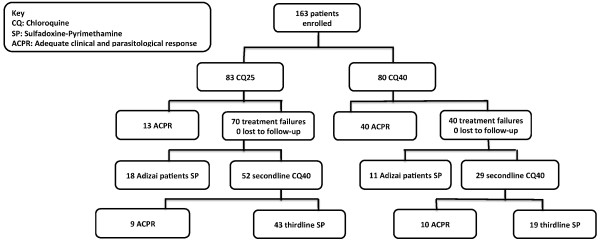
**Trial profile**.

Two trials, completed in 1998, were conducted in Baghicha, Kagan and Adizai refugee camps (Figure 1). In Baghicha and Kagan, 121 patients were recruited into two treatment groups and followed for 60 days. The 60-day duration was deliberate to allow sufficient time for back-to-back 30-day *in vivo *studies (i.e. sufficient time for cases to recrudesce following initial CQ treatment and recrudesce again following second-line CQ treatment). In Adizai camp, 21 patients were recruited per treatment group and followed for only 28 days. CQ25 patients received standard three-day treatment (CQ 25 mg/kg as 10 mg/kg on Day 0 and 1, and 5 mg/kg on Day 2). CQ40 patients received extended 5-day treatment (CQ 40 mg/kg as 10 mg/kg/day on Days 0-2 and 5 mg/kg/day on Days 3-4). Dosages were measured in 1/4 CQ tablets of 37.5 mg each to give an average dosage (range) of 26.2 (25.0, 27.8) mg/kg for the CQ25 arm and 42.1 (40.0, 45.3) mg/kg for the CQ40 arm. All treatment was directly observed for 30 minutes post-treatment.

If parasites reappeared during the follow-up period, patients received CQ40 second-line rescue treatment as per MoH and UNHCR guidelines. If parasites reappeared a further time, patients received single-dose sulphadoxine-pyrimethamine (S: 25 mg/kg, P: 1.25 mg/kg) or mefloquine (25 mg base/kg) treatment. SP and mefloquine tablets were manufactured by Roche. Chloroquine was manufactured by Aventis and supplied by the World Health Organization-Special Programme for Research and Training in Tropical Diseases (TDR). Samples of *P. falciparum *for genotyping analysis were taken at baseline from a clinical trial of CQ and SP conducted by the authors in Adizai camp and Jalalabad eastern Afghanistan in 2002 and 2003 (Rowland unpublished).

### Patient recruitment and follow-up

Participants were recruited through passive case detection at BHUs and active case detection in communities. Individuals with symptomatic falciparum malaria who met WHO *in vivo *selection criteria for low to moderate transmission settings were randomized to either CQ25 or CQ40 groups using randomized lists [10]. Exclusion criteria were infants under six months, pregnant women, vivax malaria co-infections, cases with other febrile illness, parasitaemia outside the range of 1000-100,000 asexual parasites/μl, or severe malaria. All patients gave informed consent. Ethical approval was provided by both UNHCR Health Committee and the London School of Hygiene & Tropical Medicine Ethics Committee. The trial was registered at the Clinical Trials website, reference number NCT01019408 [11].

Local health supervisors collected demographic and clinical information at enrolment, including weight, temperature, and symptoms. Supervisors directly observed treatment according to dosing schedules, prepared blood smears, and recorded temperature and clinical symptoms daily for the first five days, then every third day until day 28. Patients in Kagan and Baghicha were additionally observed every four days until day 60.

Thick and thin blood smears were stained with 3.5% Giemsa solution and all slides read on day of collection by a BHU-based microscopist. Trophozoites and gametocytes were counted against 200 white blood cells (WBC) from thick blood smears, assuming a WBC count of 8,000/μl. A smear was declared negative if no parasites were seen after examining 100 fields. Slides were re-examined for accuracy of diagnosis and recounted by an independent senior microscopist, blinded to patient, follow-up day, original result, and outcome. Differences in count were on average no greater than 5%. Finger-prick blood samples (~200 μL) were dried on Whatman filter paper prior to treatment (Day 0) and sent to LSHTM for genetic analysis.

Trial outcomes were treatment failure rates, fever clearance times (FCT), parasite clearance times (PCT), and number of recrudescences. Therapeutic responses were early treatment failure (ETF), late treatment failure (LTF) and adequate clinical and parasitological response (ACPR) using standard WHO *in vivo *criteria [10]. Parasitological responses were classified using the WHO S-RIII scale for comparison with earlier literature from low transmission settings [10,12].

### Statistical analysis

Data was double-entered in Microsoft^®^Excel, with range and consistency checks to reduce transposition error, and analysed using Stata^®^11.0. Analysis was conducted on an intention-to-treat basis. Data from the three study sites was combined for the first 28 days to calculate therapeutic outcomes and analyse subsequent malaria episodes and Kaplan-Meier survival estimates [10]. Data recorded over 60 days from Baghicha and Kaghan was used to estimate second-line therapeutic outcomes. A p-value of < 0.05 was considered significant. Univariate analysis used Pearson's chi-square (χ^2^) tests for proportions and Mann-Whitney U tests for continuous data. Logistic regression was used to calculate odds ratios (OR) of treatment success at weekly intervals and differences between treatment outcomes. *A priori *confounders (i.e. camp, gender, weight, age) were adjusted for in multivariate analysis.

### Genetic characterisation

PCR genotyping could not be conducted on patient data to determine recrudescences as samples were lost in transit. However, the authors were able to analyse 90 blood samples from falciparum cases in Adizai (the same camp) and 180 from Jalalabad, Afghanistan, collected shortly afterwards for a clinical trial to characterize resistance genotypes (Rowland unpublished). Parasite DNA was extracted from 270 blood spots collected on filter paper pre-treatment (Day 0) as described elsewhere [13]. PCR-sequence specific oligonucleotide probe assays were used to analyse genetic polymorphism of *P. falciparum *chloroquine-resistance transporter gene (*pfcrt)*at codons 72-76 and *P. falciparum *multidrug resistance protein-1 (*pfmdr1*) at codons 86 and 184 [14]. CQ resistance is associated primarily with point mutations in *pfcrt *leading to a lysine to threonine change at codon 76 (K76T) while *pfmdr1 *N86Y and Y184F are thought to have a modulatory effect [15,16]. The *pfcrt *76T and *pfmdr1 *86Y alleles may serve as predictive markers for CQ resistance in non-immune individuals living in low-transmission areas, while combined *pfcrt *76T and *pfmdr1 *86Y may be useful molecular markers for resistance to additional drugs, such as amodiaquine (AQ) [17-19].

## Results

### Enrolment characteristics

Figure 1 shows the trial profile. Of 163 patients recruited, 83 were randomized to CQ25 and 80 to CQ40 treatment groups. Table 1 shows no significant differences in enrolment characteristics between treatment groups on Day 0.

**Table 1:** Enrolment characteristics on day 0, by treatment group

Demographic characteristics	CQ 25 mg/kg	CQ 40 mg/kg
Number enrolled	83	80
Camp		
Adizai	21	21
Baghicha	44	47
Kaghan	18	12
Mean age in years (SD)	12.9 (11.3)	12.9 (11.3)
Age group		
0-5	19	18
6-14	44	44
15+	20	18
Total female	40	48
Mean weight in kg (SD)	30.1 (16.1)	32.2 (18.5)

**Clinical characteristics**	**CQ 25 mg/kg**	**CQ 40 mg/kg**

Number (%) with temperature > 37.5°C	37 (45)	42 (53)
Trophozoite density* (range)	5702 (4297-7566)	6320 (4816-8295)
Number (%) with gametocytes	38 (46)	28 (35)
Gametocyte density* (range)	140 (82-238)	148 (84-260)

NB: *refers to geometric mean. SD is standard deviation.

### First-line therapeutic outcomes

No participants were lost to follow up by day 28. Table 2 shows therapeutic and parasitological outcomes using: (a) the WHO *in vivo *system of early and late treatment failure or adequate clinical and parasitological response, and (b) the parasitological response system of S, RI, RII, RIII [10,20]. Fever clearance and parasite clearance times were similar in CQ25 and CQ40 arms (Table 2). CQ25 provided adequate clinical and parasitological response (ACPR) in only 13/83 (16%) of patients by day 28, while CQ40 provided 40/80 (50%) ACPR (adjusted OR 0.17; 95%CI 0.08, 0.38). There were few (7%) early treatment failures in either treatment group.

**Table 2:** Outcomes on day 28 by treatment group (odds ratios adjusted for age, weight, gender, and camp using logistic regression).

Outcomes	CQ 25 mg/kg	CQ 40 mg/kg	OR^1 ^(95%CI)
Total enrolled	83	80	
Total lost, excluded, or withdrawn	0	0	
Mean days to fever clearance^2 ^(95% CI)	2.6 (1.9, 3.3)	2.6 (1.9, 3.3)	
Mean days to parasite clearance (95% CI)	2.9 (2.6, 3.1)	3.1 (2.8, 3.3)	
			
**Treatment outcomes: n = 163 (%)**			
Adequate clinical and parasitological response	13 (16)	40 (50)	1
Early treatment failure	6 (7)	6 (7)	0.28 (0.07, 1.09)
Late treatment failure***	64 (77)	34 (43)	0.16 (0.07, 0.35)
			
**Parasitological outcomes: n = 163 (%)**			
S	13 (16)	39 (49)	
RI	60 (72)	40 (50)	
RII	10 (12)	1 (1)	
			
**First-line treatment success: n = 52 (%)**			
Complete parasitological cure without recrudescence***	13 (16)	39 (49)	0.17 (0.08, 0.38)
			
**First-line treatment failures: n = 111 (%)**			
Age group			
0-5	17 (89)	11 (61)	0.18 (0.03, 1.05)
6-14**	38 (86)	24 (55)	0.17 (0.06, 0.50)
15+*	15 (75)	6 (33)	0.17 (0.04, 0.70)
Gender			
Male**	35 (81)	15 (47)	0.17 (0.06, 0.51)
Female**	35 (88)	26 (54)	0.19 (0.07, 0.56)
Camp			
Adizai	17 (81)	11 (52)	0.26 (0.06, 1.12)
Baghicha**	37 (84)	24 (51)	0.16 (0.06, 0.46)
Kagan	16 (89)	6 (50)	0.23 (0.03, 1.63)

**NB: **^1^OR is odds ratio, comparing CQ40 to CQ25 adjusted for age, weight, gender, and refugee camp using logistic regression. ^2^FCT (< 37.5C) excludes those without fever on admission. *< 0.05,**p < 0.01,***p < 0.001

CQ40 patients had fewer recrudescences than did CQ25 patients during the first 28 days. Among CQ40 patients, only one recrudescent episode occurred before Day 7. Among CQ25 patients, 86% of recrudescence occurred between days 7 and 28 post-treatment. The parasitological failure rate was negatively associated with age, with failure highest among under-fives and lowest among over-fifteens (Table 2). Within each age band, failure rates were consistently lower in the CQ40 group than in the CQ25 group, irrespective of gender or camp.

**Figure 2:**
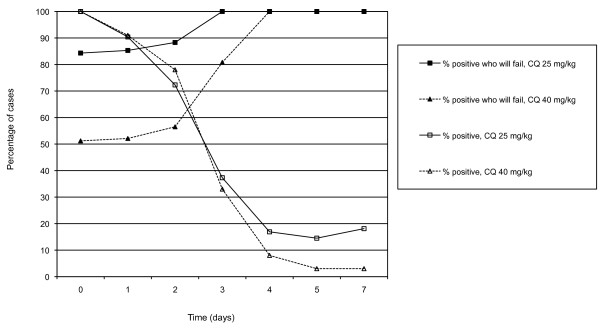
**Parasite clearance rates and probability of treatment failure among cases still positive on daily intervals after treatment start**.

Figure 2 shows the proportion of patients found positive during the first seven days of treatment and the probability of failure among those still positive on subsequent days. The longer a patient took to clear parasites the greater the probability of eventual recrudescence. All cases treated with CQ25 that were still positive on Day 3 ultimately recrudesced, while all cases treated with CQ40 still positive on Day 4 ultimately recrudesced.

**Figure 3:**
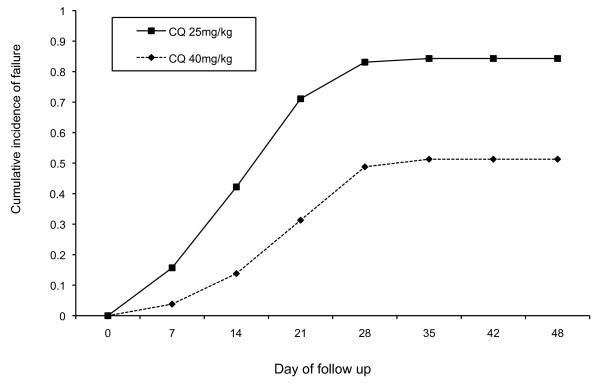
**Cumulative incidence of treatment failure for each treatment group**.

Figure 3 shows cumulative incidence of failure during each week of follow up. Table 3 shows that adjusted odds of treatment failure remained consistent between CQ40 and CQ25 groups at each 7-day interval. After Day 30 there was no further recrudescence in either group (Figure 3).

**Table 3:** Odds ratios of treatment success at weekly intervals post-treatment, comparing cq 40 mg/kg with cq 25 mg/kg, adjusted for age, weight, gender, and camp using logistic regression.

Days after treatment start	Odds ratio (95% CI)	p-value
7	0.20 (0.05, 0.73)	0.02
14	0.18 (0.08, 0.40)	< 0.0001
21	0.15 (0.07, 0.31)	< 0.0001
28	0.18 (0.09-0.38)	< 0.0001
35	0.18 (0.08, 0.39)	< 0.0001

**NB: **OR compares CQ40 to CQ25, adjusted for age, weight, refugee camp, and gender using logistic regression.

**Figure 4:**
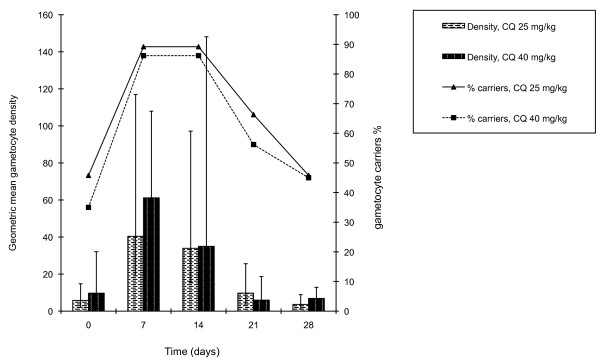
**Percentage of cases gametocytaemic and geometric mean gametocyte density (95%ci) at weekly intervals post-treatment**.

Figure 4 shows the proportion of cases gametocytaemic and average gametocyte densities at weekly intervals. There was no significant difference in the proportion gametocytaemic or in geometric mean gametocyte density between the two treatment groups at any stage after treatment.

### Second and third-line therapeutic outcomes

Table 4 provides therapeutic results for second-line treatment. CQ 40 mg/kg administered as second-line was less effective than as first-line CQ, regardless of whether first-line treatment was CQ25 or CQ40. Second-line CQ40 cured a higher proportion in the former CQ40 group than in the former CQ25 group, but this difference was not significant (adjusted OR 0.41; 95%CI 0.14, 1.19; p = 0.10). SP administered as third-line provided 88% (44/50) parasitological cure before the trial ended at 60 days.

**Table 4:** Parasitological outcomes among 81 treatment failures receiving second-line cq 40 mg/kg categorized by initial treatment group (odds ratios adjusted for age, weight, gender, and camp using logistic regression and 29 first-line failures from adizai excluded from analysis as these were treated with sp).

Outcomes	CQ 25 mg/kg	CQ 40 mg/kg	Odds Ratio (95%CI)
Total receiving CQ40 second-line treatment	52	29	
Lost, excluded	0	0	
			
**Treatment outcomes: n = 81 (%)**			
Adequate response	9 (17)	10 (34)	1
Early failure (0-3 days)	6 (12)	3 (10)	0.46 (0.09, 2.40)
Late failure (4-28 days)	37 (71)	16 (55)	0.41 (0.13, 1.20)
			
**Second-line treatment success: n = 19 (%)**			
Complete parasitological cure (0-28 days)	9 (17)	10 (34)	0.41 (0.14, 1.19)
			
**Second-line treatment failures: n = 62 (%)**			
Age group			
0-5	14 (93)	5 (62)	0.12 (0.01, 1.50)
6-14	21 (84)	12 (75)	0.77 (0.14, 4.21)
15+	8 (67)	2 (33)	0.27 (0.03, 2.45)
Gender			
Male	18 (82)	8 (67)	0.44 (0.09, 2.24)
Female	25 (83)	11 (64)	0.37 (0.09, 1.46)
Camp			
Baghicha	29 (78)	15 (65)	0.52 (0.16, 1.66)
Kagan	14 (93)	4 (67)	0.14 (0.01 2.05)

### PCR analysis of genetic markers

Table 5 shows the frequency of *pfcrt *and *pfmdr1 *point mutations among isolates from Adizai and Jalalabad. The chloroquine resistance-associated *pfcrt *codon 72-76 haplotype SVMNT (Ser-Val-Met-Asn-Thr) was present in 100% of samples successfully analysed from Adizai (63) and Jalabad (179). *Pfmdr1 *86Y was found in 14% (12/88) of Adizai and 22% (33/151) of Jalalabad samples. The *pfmdr1 *184Y allele was found in 27% (22/82) of Adizai samples and 46% (69/151) of Jalalabad samples.

**Table 5:** Pcr results for refugee isolates collected at baseline in adizai and jalalabad sites

Gene	Allele	Adizai isolates		Jalalabad isolates	
		n = 90	(%)	n = 180	(%)
*Pfcrt*	76 K	0	(0)	0	(0)
	76 T	63/63	(100)	179/179	(100)
					
*Pfmdr1 *	86 N	76/88	(86.4)	118/151	(78.1)
	86 Y	12/88	(13.6)	33/151	(21.9)
					
	184 Y	22/82	(26.8)	69/151	(45.7)
	184 F	60/82	(73.2)		

NB: Denominators are all readable isolates. The Jalalabad isolates of 184F could not be read.

## Discussion

Chloroquine failure rates were higher than anticipated, and since administration was directly observed, failure was due to resistance rather than poor adherence. Analysis showed that with 51% failure in CQ40 and 84% failure in CQ25, chloroquine is no longer suitable for falciparum malaria treatment among Afghan refugees, either as first or second-line with short or extended regimens, and usage should stop. Second-line CQ40 achieved a higher failure rate than did first-line, demonstrating lack of suitability for this purpose.

While the authors were unable to make use of PCR genotyping to distinguish recrudescent from new infections, other *in vivo *trials conducted in the area that did include genotyping indicated fewer than 5% would be new infections [21]. The most compelling evidence for subsequent infections being therapeutic failures is the very high failure rates by Day 28 and apparent absence of any new parasitaemias over 60 days of follow up.

The finding of the *pfcrt *76T mutation in 100% of isolates analysed is consistent with a low degree of heterogeneity in the parasite population, as also shown by Khatoon et al in isolates from nearby Bannu district NWFP [15]. The *pfcrt *76T allele is strongly associated with CQ and amodiaquine (AQ) resistance in falciparum isolates from Asia, Papua New Guinea, Africa, and South America [9,15]. *Pfmdr1 *86Y and 184Y alleles, which are also associated with CQ and AQ resistance, were present in only a minority of our isolates from Adizai camp or from Bannu district [15]. In a clinical trial of AQ in nearby Afghanistan, which also resulted in high rates of recrudescence, *pfmdr1 *alleles were not strongly selected among treatment failures [22]. These findings indicate that the *pfcrt *codon 72-76 haplotype SVMNT present in Pakistan is sufficient by itself (i.e. without *pfmdr1 *86Y and 184Y) to cause high-level CQ and AQ resistance [9,22]. By contrast, in Africa where the CQ-resistant variant *pfcrt *codon 72-76 CVIET appears to be the predominant haplotype, AQ remains relatively effective [9]. In the one clinical trial in East Africa where AQ did demonstrate high levels of *in vivo *resistance, the CQ-resistant variant CVIET haplotype was present with *pfmdr1 *86Y and 184Y alleles which presumably added to the resistance there [9,23-26].

The rate of parasitological failure was higher after second-line than after first-line CQ40 treatment. Recrudescent infections presumably started with higher proportions of resistant parasites than did initial infections. However, this cannot explain why the initial CQ40 course seemed to eliminate around 39% of resistant infections, as indicated by the improved cure rates over 60 days following initial five-day (51% recrudescence) as compared to three-day treatment (84% recrudescence). Among this 39%, any resistant parasites must have been removed by the additional two days of treatment and did not reappear over the subsequent 60 days. Ursing *et al *have, in parallel, undertaken clinical studies with high-dose CQ in Guinea-Bissau [27,28]. They found that high-dose CQ (75 mg/kg as split-dose over five days) was well-tolerated (as was the 40 mg/kg administered in the present trial) and 78% of infections carrying *pfcrt *76T were successfully treated compared to only 34% with 25 mg/kg [27]. This was a higher treatment success rate than in Pakistan.

While *pfcrt *76T was highly prevalent in the Pakistan samples, *pfcrt *76T prevalence in the Guinea-Bissau population, discussed above, remained stable at a much lower 25% between the years 1990 and 2005 [27,29]. These contrasts in *pfcrt *76T between continents are likely due to differences in the fitness of resistance alleles, as the *pfcrt *72-76 SVMNT resistance haplotype dominant in India, Iran, Pakistan and Afghanistan is not associated with re-emergence of CQ sensitivity or fluctuations in seasonal prevalence shown by the CVIET haplotype in some parts of Africa [27,28,30-32]. Drug pressure may also affect stability. If most infections were treated with a quinoline, *pfcrt *76T frequency would remain high. In the African settings, sensitive parasites may have found a niche in the many untreated infections, where their greater fitness would allow them to compete better than any co-infecting resistant parasites. It has been more difficult for CVIET-carrying parasites to gain the same high prevalence in Africa as SVMNT-carrying parasites have achieved in parts of Asia.

The present trial did not measure adherence, as it was designed to assess efficacy rather than effectiveness. Consequently, it cannot challenge the initial assumption that refugees fail to adhere to either three-day or five-day courses. However, recent research demonstrates that with appropriate instructions - as in a trial of unsupervised 14-day primaquine treatment - Afghan refugees will adhere to much longer treatment regimens than the five-day course described here [22,33]. What is clear from directly-observed treatment is that neither CQ25 nor CQ40 regimens can be justified, as neither can provide acceptable cure rates given the high prevalence of CQ-resistant falciparum malaria now in Pakistan [4,5].

In Afghanistan, where many refugees have returned, most malaria cases are still treated with CQ, without parasitological diagnosis by either microscopy or rapid diagnostic test [34]. This makes the present study, though conducted more than ten years ago, still highly relevant. About 70-90% of cases in Afghanistan are due to vivax and will respond to CQ. However, most of the falciparum cases treated with CQ - whether for three days or longer - are likely to fail [33]. While the total number of falciparum cases will be small, without effective treatment these risk developing into severe malaria. As up to one-fifth of suspected malaria cases arrive at government clinics with detectable chloroquine present in their urine, irregular or intermittent treatment with chloroquine might be common [35]. The need for routine parasitological diagnosis by microscopy or rapid diagnostic test to allow differential and, most importantly, effective treatment for both falciparum and vivax malaria is paramount in Pakistan and Afghanistan.

Although combination therapy using artesunate-SP has been adopted as policy for treatment of confirmed falciparum malaria in Pakistan and Afghanistan, implementation is patchy and uptake slow [21]. While follow-up and numbers of SP patients were too few in this trial to determine significance, the 12% SP failure rate raises questions about SP's long-term efficacy. While SP has a role as combination partner in Pakistan and Afghanistan, if administered without artesunate, resistance to SP may select rapidly [36,37].

## Competing interests

The authors declare that they have no competing interests.

## Authors' contributions

NH analysed and interpreted data and drafted the manuscript. ND was responsible for data collection, PCR analysis, and critically reviewing the manuscript. SS assisted with initial analysis and critically reviewed the manuscript. RH and KB provided critical analysis of genotyping results and critically reviewed the manuscript. MR conceived and designed the study, and revised the manuscript critically for intellectual content. All authors approved the final version for publication.
